# Interpersonal Perceptions of Adverse Peer Experiences in First-Grade Students

**DOI:** 10.3389/fpsyg.2018.01165

**Published:** 2018-07-10

**Authors:** Francisco J. García Bacete, Inmaculada Sureda-García, Victoria Muñoz-Tinoco, Irene Jiménez-Lagares, Ghislaine Marande Perrin, Jesús F. Rosel

**Affiliations:** ^1^Department of Developmental, Educational and Social Psychology, and Methodology, Jaume I University, Castellón de la Plana, Spain; ^2^GREI Interuniversity Research Group, Jaume I University, Castellón de la Plana, Spain; ^3^Department of Applied Pedagogy and Educational Psychology, University of the Balearic Islands, Palma de Mallorca, Spain; ^4^Department of Developmental and Educational Psychology, University of Seville, Seville, Spain

**Keywords:** adverse peer experiences, negative interpersonal perceptions, peer victimization, mutual antipathy, peer rejection, peer social system

## Abstract

**Aim:** The aim of this study was to identify which adverse peer experiences better predict perceived negative peer relationships among elementary school first graders according to sex. The peer experiences examined were peer rejection, peer victimization, and mutual antipathy; the interpersonal perceptions studied were perceived peer victimization, dyadic meta-perception of peer disliking, and loneliness.

**Methods:** The participants were 809 children (*M*_age_ = 6.4 years, *SD* = 0.32; *n*_girls_ = 412, 50.9%) enrolled in 35 first-grade classes from 15 schools in 4 Spanish regions: Valencia, *n* = 276, 34.1%; Balearic Islands, *n* = 140, 17.3%; Andalusia, *n* = 199, 24.6%; Castile-Leon, *n* = 194, 24%. We calculated sex differences in peer experiences and interpersonal perceptions by means of one-way ANOVA for means differences and Fisher’s *r*-to-*z* transformation for correlations differences. We used a multilevel regression analysis (nesting variables: class and region) to determine whether the associations between each peer experiences and each perception were unique.

**Results:** Each adverse peer relationship predicted each interpersonal perception differentially. Peer victimization was a good predictor of the three interpersonal perceptions, and the only predictor of perceived peer victimization. Peer rejection predicted loneliness, whereas mutual antipathies predicted dyadic meta-perception of peer disliking, although more so among girls. A significant effect at region level was found but not at class level.

**Conclusion:** Our findings suggest that research should take into account the different levels of the social peer system when analyzing peer experiences within the classroom context. The study contributes to sensitize teachers about the greater responsiveness of 6-year-old girls to adverse peer experiences, and it could be useful for designing interventions that would help children oppose rejection and empower active bystanders to fight against peer mistreatment.

## Introduction

When initiating elementary education, the number of peer interactions rises considerably (Rubin et al., 2006). During this period, the presence of negative social relationships also increases. Together with peer rejection, other negative social interactions that were incipient in early infancy emerge during this stage, such as peer victimization (Leadbeater and Sukhawathanakul, 2011) and mutual antipathy (Card, 2010). Peer victimization is the experience of being the target of any form of aggressive attack (Salmivalli and Peets, 2009). Two people are considered to be immersed in a mutually antipathetic relationship when there are reciprocal negative feelings between them (Abecassis, 2003). Peer rejection is a covert phenomenon that occurs when a significant number of members in a group have negative feelings toward a group member (McDougall et al., 2001; Leary, 2005). Peer victimization, mutual antipathy, and peer rejection are frequent adverse experiences in childhood and adolescence (Huitsing et al., 2012). About 5–10% of school children are chronically victimized (Schuster, 2001; Juvonen and Graham, 2014), about 35% have at least one mutually antipathetic relationship during their childhood or adolescence (Card, 2010), and about 13–16% are rejected by peers (McKown et al., 2011).

Peer rejection, peer victimization, and, to a lesser extent, mutual antipathy were associated with externalizing problems, such as aggressive, antisocial behavior and school difficulties, and internalizing problems, such as low self-esteem, anxiety, and depression (McDougall et al., 2001; Bukowski et al., 2007; Card, 2010; Kamper-DeMarco and Ostrov, 2017).

The symbolic interactionism approach permits studying children’s interpersonal perceptions conceptualized as one’s self-understanding that results from social exchanges (Cillessen and Bellmore, 2004). Research findings suggest that negative perceptions of their peer experiences are a risk factor for maladjustment (Guerra et al., 2004; Salmivalli and Isaacs, 2005). It seems therefore of utmost interest to study which adverse peer experiences make children form negative interpersonal perceptions and, when adverse experiences are concurrent, what the contribution of each one is in the presence of the other experiences.

### Peer Social System

Children’s experiences with peers can be best understood by referring to processes of different levels of social complexity – social interactions, dyadic relationships, and group processes – that occur given particular contributions of individual characteristics and cultural features (Rubin et al., 2006). Interaction level refers to the social exchanges of some duration in which the participants’ behaviors are interdependent, like, for instance, peer sociability and peer victimization. Dyadic relationships are characterized by the reciprocity of affection between the two members of the dyad. Best friend and mutual antipathy belong to this level. A group is the structure that emerges from the features and patterning of the interactions and relationships present in a population of children (e.g., a classroom). Peer rejection and acceptance are examples of group processes. Although the constructs and processes at a particular level are conceptually distinct from those at other levels, they are interdependent in the sense that constructs at one level can be constrained or influenced by constructs at other levels (Rubin et al., 2015). Studies on peer relationships integrating the several social levels can help understanding their influence on interpersonal perceptions (Zhang et al., 2014).

Taking the peer system proposed by Rubin et al. (2006, 2015) as reference, we have selected for this study three adverse peer experiences: peer victimization, mutual antipathy, and peer rejection. Each adverse peer experience matches one of the levels and each one is distinct from the others but is also associated with the others in some way (Boivin et al., 2001; Schuster, 2001; Leary, 2005; Card, 2010). Victimization is a social interaction or behavioral level, mutual antipathy is a dyadic relationship, and rejection is a group phenomenon. Furthermore, whereas rejection and mutual antipathy may be limited to covered peer sentiments, victimized children experience negative behaviors in addition to negative peer attitudes (Schuster, 2001). Victimization is an abusive interaction (Ladd et al., 1997; Schuster, 2001), whereas mutual antipathy is a relationship of negative reciprocity between the two parties involved (Abecassis, 2003). Peer victimization, peer rejection, and mutual antipathy are concurrent experiences in childhood (Schuster, 2001; Ladd and Troop-Gordon, 2003; Card, 2010; Godleski et al., 2015), which arouses the interest in studying these experiences simultaneously.

Moreover, each child has person-related characteristics that they bring to and take away from their experiences with peers, such as their sex – an issue that we address later – and their interpersonal perceptions. Interpersonal perception refers to one’s understanding of oneself in social interactions and of how one thinks that the others see him/her (Cillessen and Bellmore, 2004). Interpersonal perceptions result from social experiences that occur at different levels. In this line, authors have differentiated between specific, dyadic, and generalized interpersonal perceptions (Bellmore and Cillessen, 2003; Cillessen and Bellmore, 2004; Calhoun, 2011). Specific perception refers to individuals’ knowledge of how they are the recipient of specific behaviors by peers (e.g., “My peers call me names”). Dyadic meta-perception refers to individuals’ knowledge of how they are seen by particular peers (e.g., “Who likes you the least?”). Generalized perception refers to individuals’ knowledge of how they see themselves in peer interaction in a general way (e.g., “It is hard to get kids in school to like me”).

Given that different interpersonal perceptions focus on different peer levels, to study simultaneously perceptions of experiences that occur at different peer levels would be useful for understanding the link between perceptions and experiences. In this sense, together with the three aforementioned adverse social experiences, we have selected three negative interpersonal perceptions belonging to the three previously described types. Perceived peer is a perception of being the target of specific negative behaviors by peers (Calhoun, 2011) that informs of feelings of being victimized by peers. Dyadic perceived peer disliking (henceforth dyadic perceived disliking) is a dyadic meta-perception in which a child names the particular peers by whom she/he perceives herself/himself to be rejected (Bellmore and Cillessen, 2003); it informs of feelings of being rejected by particular peers. Loneliness is a cognitive awareness of a generalized deficiency in one’s social and personal relationships (Asher and Paquette, 2003) that informs of feelings of being alone at school.

Finally, each of these levels falls under the all-reaching umbrella of the one cultural macrosystem (Bronfenbrenner and Morris, 2006), which implies that the same social behaviors or relationships have different psychological meaning from one culture to another. In this study, we performed an exploratory approach of the influence of cultural level by selecting schools of four Spanish regions that respond to culturally different values or stereotypes, namely Castilian, Andalusian, Balearic, and Valencian.

In the two following sections, we reviewed the literature, first, on the associations between the adverse experiences and perceptions selected for this study, and then, on the influence of sex in such associations.

### Association Between Adverse Peer Experiences and Negative Interpersonal Perceptions

The literature reports that different adverse peer experiences are associated in similar ways with subjective distress and negative interpersonal perceptions (e.g., low self-esteem, loneliness, anxiety, unhappiness, exclusion, and insecurity among peers) (Boivin and Hymel, 1997; Sandstrom and Zakriski, 2004; Card, 2010). However, those studies failed to take into account that these adverse experiences are usually present simultaneously (Ladd et al., 1997) and that, although as seen above, they differ in nature, their effects can overlap (Bukowski and Hoza, 1989).

Adverse peer experiences have usually been studied separately or two at a time (e.g., Ladd and Troop-Gordon, 2003). Exceptions are the works by Ladd et al. (1997) with a sample of 5- to 6-year-old children, Boivin and Hymel (1997) with 8- to 10-year olds, and Salmivalli and Isaacs (2005) with 11- to 13-year olds, which studied simultaneously peer victimization, peer rejection, and friendlessness in relation to children’s interpersonal perceptions. Yet, in these studies, only one or two perceptions were examined, namely perceived acceptance and loneliness, which both focus on the group level. This might explain why neither of these two perceptions was predicted by the friendlessness measure used, regardless of whether it was number of friends, number of affiliations, or having or not having a friend. Such results suggest the inclusion of other perceptions that would focus on the interactional and dyadic levels of the peer system, as we do in this work. To the best of our knowledge, mutual antipathy has not been studied before together with victimization and rejection. However, such study is of interest, considering that friendlessness is characterized by the absence of positive reciprocity whereas mutual antipathy features the presence of negative reciprocity.

#### Perceived Peer Victimization

There is a broad consensus in the literature that children perceive victimization when they are subjected to interactions of peer victimization (Boivin and Hymel, 1997; Juvonen and Graham, 2014). Regarding peer rejection, rejected children tend to report more perceptions of victimization than non-rejected children (Kärnä et al., 2010). Peer rejection puts a child at risk of being harassed by peers, but it is precisely this type of manifest behavior that makes the child perceive themselves to be victimized (Boivin et al., 2001). In a similar way, Abecassis (2003) observed that some children with mutual antipathies tended to perceive the behavior of the other member of the relationship as a threat toward themselves. Again, it is the behavior of others that leads to feeling victimized, rather than the condition of mutual antipathy itself.

#### Dyadic Perceived Disliking

Two works have examined dyadic perceived disliking as dependent variable, with disparate results. MacDonald and Cohen (1995) found that rejected children were more aware of their negative peer relationships than accepted children, whereas Cillessen and Bellmore (1999) did not find any differences. Studying generalized perceived rejection as dependent variable, Ladd and Troop-Gordon (2003) found that exposure to peer rejection undermined children’s feelings of being accepted by their peers and by themselves in the future. Yet, rejected children who are not treated aversively by their peers may not be aware that they are disliked (Boivin and Hymel, 1997; Boivin et al., 2001). For rejected children who are not overtly disliked, dyadic relationships like friendlessness or mutual antipathy are likely to be a more salient aspect of their daily peer experiences than group dynamics (Sandstrom and Zakriski, 2004). According to Card (2010), mutual antipathy implies a personal and particularly intense feeling of social rejection.

#### Loneliness

Victimized children experience more loneliness than children who are not victimized (Ladd et al., 1997; Boivin et al., 2001; Asher and Paquette, 2003; Catterson and Hunter, 2010; Morrow et al., 2014). Mutual antipathy is also positively associated with loneliness feelings, though with small correlations (Card, 2010). As for peer rejection, it is reliably associated with feelings of loneliness and other emotional distress (Boivin et al., 2001; Asher and Paquette, 2003; Zhang et al., 2014). According to Sandstrom and Zakriski (2004), peer rejection should play a causal role in the emergence of these adjustment difficulties, which would place it at the core of the correlates of loneliness.

### The Role of Sex in the Association Between Adverse Peer Experiences and Negative Interpersonal Perceptions

Boys usually report more adverse experiences than girls, but this is not always so. For instance, regarding peer victimization, although boys often score higher on direct physical victimization than girls (Paquette and Underwood, 1999), such differences in relational victimization are less consistent (Rose and Rudolph, 2006; Underwood, 2007; Bevans et al., 2013), and some studies found no sex differences (Rose and Rudolph, 2006). On the contrary, although girls usually report more stress with peers than boys do (Rose and Rudolph, 2006), some studies found no sex effects of adverse peer experiences on loneliness and perceived peer acceptance (Ladd et al., 1997; Salmivalli and Isaacs, 2005). In the same vein, the research on sex differences in the association between negative experiences and negative interpersonal perceptions reports mixed results, as seen below.

First, concerning perceived peer victimization, research found sex differences when focusing on the intention of the victimization. Peer victimization may be more distressing for girls when it aims to damage relationships, whereas it is more upsetting for boys when it attempts to challenge strength and dominance (Underwood, 2007; Morrow et al., 2014). In line with these results, Paquette and Underwood (1999) observed that girls were more sensitive to situations of social exclusion or peer rebuff than to other forms of victimization that did not entail loss of relationships.

Second, with regard to dyadic perceived disliking, considering that girls tend to interact in dyadic contexts more than boys and report more forms of friendship stress, Burks et al. (1995) conjectured that, for girls, the lack of close relationship with a friend is more severe than rejection by the large peer group. However, Cillessen and Bellmore (1999) did not find any sex difference in perceived rejection at dyadic or at group level, in any of the group status.

Third, sex effects on loneliness are not conclusive either. According to McDougall et al. (2001), girls appear to be more likely to isolate themselves and experience loneliness in reaction to peer rejection and peer victimization, whereas Boivin and Hymel (1997) found a small interaction effect of peer rejection on loneliness only for boys. Finally, Asher and Paquette (2003) reported that sex differences in loneliness were rarely significant.

As seen, previous studies on the association between sex and negative interpersonal perceptions have presented mixed results, probably because those studies did not take into account that girls are involved in less adverse peer experiences than boys. Based on the symbolic interaction, one would expect girls to experience fewer negative interpersonal perceptions than boys. The fact that research findings do not show differences in negative perceptions or that negative perceptions are higher in girls indicates that girls, according to the socialization model, value friendship and common goals more than boys do. Girls might experience more stress in situations where common goals are not achieved or that involve loss of relationships with peers (Rose and Rudolph, 2006).

### Objectives and Hypotheses

The present study intends to respond to the following research questions: (a) Which adverse peer experiences better predict perceived negative peer relationships among elementary school first graders?, and (b) Is there any sex difference in the predictions?

The first objective was to determine the contribution of each one of the frequent, concurrent, and diverse adverse peer experiences (peer victimization, mutual antipathy, and peer rejection) to each one of the three different negative interpersonal perceptions (perceived peer victimization, dyadic perceived disliking, and loneliness), when all three adverse peer experiences were present simultaneously. In light of the afore-reviewed literature, we expected all the adverse experiences to predict all the interpersonal perceptions (Sandstrom and Zakriski, 2004), overlapping their effects (Bukowski and Hoza, 1989). However, we expected a peer experience taking place at a certain social level to have a stronger association with the self-perception focusing on the same level, as both are conceptually more related (Rubin et al., 2015). Thus, our first hypothesis was that perceived peer victimization would most likely be linked to peer victimization because of the interactional/behavioral nature, level, or focus of both (Rubin et al., 2006; Calhoun, 2011). The second hypothesis was that perceived disliking would mostly associate with mutual antipathy because of the dyadic nature of both (Bellmore and Cillessen, 2003; Rubin et al., 2006). The third hypothesis was that loneliness would more robustly relate to peer rejection because of the generalized or group nature of both (Bellmore and Cillessen, 2003; Rubin et al., 2015). Notwithstanding the foregoing, as children’s self-perceptions stem above all from the social behaviors peers direct toward them (Hymel et al., 1999), as fourth hypothesis we expected peer victimization to be heavily present in the link with all the self-perceptions analyzed.

The study’s second objective was to determine whether there were sex differences (individual differences) in the association between adverse peer experiences and negative interpersonal perceptions, as there should be according to the socialization model (Rose and Rudolph, 2006). As seen above, girls seem to be more sensitive than boys to negative dyadic and group experiences (Burks et al., 1995; Paquette and Underwood, 1999; McDougall et al., 2001). Thus, in the above formulated hypotheses, we expected to find sex differences in interpersonal perceptions focusing on peer relationships at dyadic and group relationships.

## Materials and Methods

### Participants

We used incidental sampling to select public elementary schools representative of medium socioeconomic status, situated in urban districts close to the four universities where we were conducting a broader study that included a large intervention later on. The students were enrolled in 35 first-grade classes at 15 public elementary schools in four Spanish regions; 34.1% of the students (*n* = 276, out of whom *n*_girls_ = 136, 49.3%) attended school in Valencia, 17.3% (*n* = 140, out of whom *n*_girls_ = 71, 50.7%) in the Balearic Islands, 24.6% (*n* = 199, out of whom *n*_girls_ = 105, 52.8%) in Andalusia, and 24% (*n* = 194, out of whom *n*_girls_ = 100, 51.5%) in Castile-Leon. The number of students per classroom ranged between 18 and 26, with an average of 23.1. The children’s ethnic backgrounds were Caucasian (*n* = 736, 91%), Romany (*n* = 26, 3.2%), South-American (*n* = 16, 2%), Arabian (*n* = 14, 1.7%), Asian (*n* = 12, 1.5%), and Black (*n* = 5, 0.6%). Children’s nationality was mostly Spanish (88%); the students from other countries came mainly from South America and Eastern Europe.

Of the 809 participating students (*n*_girls_ = 412, 50.9%), 774 were informants (95.7%; 51.2% girls). The mean age of the sample was 6.4 years (*SD* = 0.32). As regards the 35 non-informant subjects, we did not have parental consent for 19, and the remaining 15 did not attend school at data collection. We found no difference in sex, ethnic background, or nationality between informant and non-informant groups, compared through chi squared difference test. Non-informant children were not included in the study, although they could be named by their peers in the peer victimization and sociometric nominations. Average rate of participation per classroom was 95.7%; the lowest rate was 80%, in two classrooms. Using unlimited nominations, stable constructs are still obtained with a 60% participation rate (Cillessen and Bukowski, 2017). Missing data in the variables ranged from 0.5 to 3.1%, lower than the 5% suggested by Tabachnick and Fidell (2014) as a limit to consider that the data may be biased.

### Ethics Statement

The study, was conducted in accordance with the 1964 Helsinki Declaration and its later amendments, with the approval of the teachers, school boards, and the Spanish competent education authorities [blinded]. Review and approval from the ethics committee of our institution, Jaume I University, was obtained. Participation was voluntary. The families provided the required written informed consent.

### Procedure and Measures

The measures were age appropriate for the six-year-old participants and administered in two separate individual interviews conducted with each child outside the classroom by a trained researcher who read the items aloud to the student. Each interview, in which the child completed two instruments, lasted 25 min.

#### Sociometric Nominations

We used a four-item sociometric questionnaire for unlimited peer nominations, proposed by García Bacete et al. (2014). We showed to each child a set of photos of their classroom peers and asked: “Who in your class do you like the most?”, “Who in your class do you like the least?”, “Who likes you the most?”, and “Who likes you the least*?*” In this study, we used only the two “*like least*” questions.

The Sociomet program (González and García Bacete, 2010) was used to calculate the following three sociometric indices: the index of Negative Nominations Received (NNR/*n*-1)^∗^100, which indicates peer rejection; the index of Negative Reciprocity or mutual negative nominations (NR/*n*-1)^∗^100, which indicates mutual antipathy; the index of Negative Nominations Expected (NNE/*n*-1)^∗^100, which indicates dyadic perceived disliking. All of the indices are percentages in which the denominator is the number of consented students in the classroom minus 1 (*n*-1). The score therefore ranges between 0 and 100. The validity of this method has been repeatedly demonstrated (Cillessen and Bukowski, 2017; García Bacete and Cillessen, 2017).

#### Peer Victimization Subscale

Victimization was evaluated using a technique known as descriptive matching, in which peers are asked to name classmates who best fit a given description. We used the four items from the Peer Victimization subscale of the Extended Class Play proposed by Burgess et al. (2003). These items inform of physical, verbal, and relational mistreatment and exclusion by others (e.g., “Someone who is hit or kicked by other kids”). We then calculated the percentage of nominations received for each subject in each item. For each student, we calculated composite scores as the average of the four items. The scores of Peer victimization ranged from 0 to 100. According to the CFA with robust method estimation, the 4 items form one factor: [χ^2^_S-B_ (1) = 2.597, *p* = 0.11; χ^2^_S-B_/df = 2.60; BBNN = 0.86; CFI = 0.98; RMSEA = 0.078, with 90% confidence interval ranging from 0.000 to 0.200]. Cronbach’s alpha, composite reliability, and average variance extracted were 0.73, 0.71, and 0.39, respectively.

#### Perceived Peer Victimization Scale

This is a self-report questionnaire consisting of eight items (García Bacete et al., 2014). We asked the participants to report the frequency (*never, rarely, quite often, almost every day*) with which, in the last month, they had experienced a situation involving victimization or hostility by peers (i.e., “Some of your classmates insult you, call you names”). Each item had a range of 1 to 4, and the Perceived Peer Victimization score for each subject was the average of the eight items comprising the factor. According to the CFA with robust method estimation, the 8 items form one factor: [χ^2^_S-B_ (20) = 33.281, *p* = 0.03; χ^2^_S-B_/df = 1.66; BBNN = 0.98; CFI = 0.99; RMSEA = 0.030, with 90% confidence interval ranging from 0.009 to 0.047]. Cronbach’s alpha, composite reliability, and average variance extracted were 0.82, 0.82, and 0.36, respectively.

#### Loneliness and Social Dissatisfaction Questionnaire

The original scale has 24 items (Cassidy and Asher, 1992; Spanish validation in García Bacete et al., 2014); 16 of these items measure loneliness and social dissatisfaction. Students indicated their level of agreement with each item by responding, *yes, no*, or *sometimes*. The two-factor model with covariance between the two factors, Loneliness and Social Dissatisfaction, showed good fit in the CFA with robust method estimation [χ^2^_S-B_ (76) = 114.79, *p* = 0.003; χ^2^_S-B_/df = 1.51; BBNN = 0.96; CFI = 0.96; RMSEA = 0.026 with 90% confidence interval ranging from 0.016 to 0.035]. For this study, only the factor that specifically evaluates loneliness was used (e.g., “Do you feel left out of things at school?”). Each item had a range of 1–3, and the Loneliness score for each subject was the average of the six items comprising the factor. Cronbach’s alpha, composite reliability, and average variance extracted were 0.68, 0.69, and 0.27, respectively.

### Data Analysis

First, descriptive statistics and Pearson’s (*r*) correlations for each sex were calculated to analyze the associations between adverse peer experiences, between negative interpersonal perceptions, and between both sets of variables. Second, we calculated sex differences in peer experiences and interpersonal perceptions, using one-way ANOVA for means differences and Fisher’s *r*-to-*z* transformation for correlations differences (Preacher, 2002).

Third, a multilevel regression analysis (Schumacker and Lomax, 2010; Heck and Thomas, 2015) was performed with each interpersonal perception as a dependent variable to determine whether associations that emerged between specific adverse peer experiences as independent variables and each perception were unique or redundant relative to other forms of peer relationships. In addition to peer experiences as antecedents, the following aspects were considered: (a) as each child belonged to a class, a random coefficient for the intercept (Level 1: child; Level 2: class) was incorporated into each analysis; (b) because schools from four different regions were included, the variable region was included as a factor (dummy variable); (c) because the children’s behavior could differ according to their sex, the interaction of sex with each peer experience (Sex^∗^Peer victimization, Sex^∗^Mutual antipathies, and Sex^∗^Peer rejection) was included, following Jose (2013). This set of antecedents forms the full model for each dependent variable.

The analytical procedure was similar to other studies on peer experiences (e.g., Ladd et al., 1997). For each dependent variable, we present, first, the intra-class correlation (ICC) and the null model (intercept only); subsequently, we formulated the complete regression model (full model), and we then simplified it with the aim of selecting the simplest one of them (fitted model). In the fitted model, only the statistically significant independent variables were retained and, regardless of whether they were significant, the random component of the multilevel classroom intercept ($S_{u}^{2}$) and the dummy region were kept because they formed part of the assumptions of the model. The -2 log likelihood Δ(-2LL) of the full and fitted models was compared with their null model to check whether each model was significant; and finally, the difference of the -2LL, Δ(-2LL) and the difference of the Akaike information criterion, Δ(AIC) were calculated to compare the nested models (full and fitted model for each dependent variable). We used the Burnham et al. (2011) procedure, which establishes that two models are different when Δ(AIC) > 7. The SPSS (2015) program was used. Visual representations of the models are provided by means of effect diagrams and figures of regression slopes.

## Results

### Descriptive Analyses

**Table 1** presents the descriptive statistics of the variables used in the study and their correlations. All correlations were positive. The correlations between peer experiences were medium size (0.32–0.65 for boys, and 0.26–0.56 for girls), which indicates that despite being related to each other, each adverse peer experience retains a part of unique variance. The correlations between the three interpersonal perceptions were small or null (0.00–0.31 for boys, and 0.10–0.32 for girls), which indicates that the three perceptions were different. Finally, significant correlations between all peer experiences and interpersonal perceptions were observed.

**Table 1:** Descriptive statistics and correlations between the study variables according to Sex.

	Boys		Girls			Boys/Girls^1^					
	*Mean*	*SD*	*Mean*	*SD*	*F*	1. PV	2. MA	3. PR	4. P-PV	5. DPD	6. L
1. Peer victimization (PV)	11.25	8.04	7.03	4.55	85.041ˆ***	–	0.26ˆ***	0.40ˆ***ˆa	0.15ˆ***d	0.19ˆ***	0.17ˆ***
2. Mutual antipathy (MA) (%)	2.89	5.19	2.26	4.03	3.578ˆ*	0.32ˆ***	–	0.56ˆ***	0.16ˆ***	0.40ˆ***c	0.06
3. Peer rejection (PR) (%)	15.63	15.24	10.49	11.32	29.850ˆ***	0.65ˆ***a	0.54ˆ***	–	0.19ˆ***	0.32ˆ***f	0.22ˆ***g
4. Perceived peer victimization (PPV)	1.90	0.74	1.86	0.71	0.782	0.28ˆ***d	0.04	0.19ˆ***	–	0.27ˆ***b	0.32ˆ***
5. Dyadic perceived disliking (DPD) (%)	10.04	9.21	9.89	9.81	0.050	0.19ˆ***	0.20ˆ***e	0.16ˆ***f	0.05ˆb	–	0.10ˆ*c
6. Loneliness (L)	1.67	0.50	1.62	0.47	1.518	0.21ˆ***	0.12ˆ**	0.19ˆ***g	0.31ˆ***	0.00ˆc	–

*^*1*^Upper part of the table: girls; Lower part of the table: boys. Differences between pairs of correlations according to Sex: ^*a,b,e,f*^*p* < 0.01; ^*c,d,g*^*p* < 0.10; *^∗^p* < 0.10; ^∗∗^*p* < 0.05; *^∗∗∗^p* < 0.01.*

#### Analyses of Variance and Correlations Differences: Sex Differences

The one-way ANOVAs informed that boys were involved in more adverse peer experiences than girls. The association between peer rejection and peer victimization was higher in boys than in girls (*r*_b_ = 0.65, *r*_g_ = 0.40, *p* < 0.01). No sex difference in negative interpersonal perceptions was found. In boys, the only significant correlation was the one between loneliness and perceived peer victimization (*r*_b_ = 0.31). The correlations were higher in girls than in boys when dyadic perceived disliking was participating (with perceived peer victimization *r*_b_ = 0.05, *r*_g_ = 0.27, *p* < 0.01; with loneliness, *r*_b_ = 0.00, *r*_g_ = 0.10, *p* < 0.10).

There were also sex differences in the correlations between peer experiences and interpersonal perceptions. The correlations of dyadic perceived disliking with mutual antipathies and with peer rejection were stronger in girls than in boys (*r*_b_ = 0.20, *r*_g_ = 0.40, *p* < 0.01; and *r*_b_ = 0.16, *r*_g_ = 0.32, *p* < 0.01, respectively) and peer rejection with loneliness (*r*_b_ = 0.19, *r_g_* = 0.22, *p* < 0.10). The association between peer victimization and perceived peer victimization was stronger in boys than in girls (*r*_b_ = 0.28, *r*_g_ = 0.15, *p* < 0.10).

#### Predictive Analyses: Adverse Peer Relationships Predicted Interpersonal Perceptions

Study objectives were to analyze how well the three peer experiences jointly predicted each interpersonal perception as a function of sex. **Table 2** presents the results of the full model and the fitted model for each perception.

#### Perceived Peer Victimization

**Table 2** shows that the ICC was very small and the $S_{u}^{2}$ = 0.005 was nonsignificant. The significant antecedent variables of perceived peer victimization (full model M1) were Peer Victimization and Region.

**Table 2:** Results of multilevel regression analysis of Perceived Peer Victimization, Dyadic Perceived Disliking, and Loneliness.

Dependent	Perceived		Dyadic perceived		Loneliness	
variable	victimization		disliking			
ICC	0.036		0.121		0.085	
Null model	1636.851,		5675.186,		1080.378,	
(-2LL)	2 parameters (Ps)		2 Ps		2 Ps	
	
	**Full model**	**Fitted model**	**Full model**	**Fitted model**	**Full model**	**Fitted model**
	**M1**	**M2**	**M3**	**M4**	**M5**	**M6**
	
	**Estimated coefficient values**					

Intercept	1.610^∗∗∗^	**1.643^∗∗∗^**	5.946^∗∗∗^	**5.887^∗∗∗^**	1.704^∗∗∗^	**1.701^∗∗∗^**
Sex	0.039	–	–0.622	–0.456	–0.060	–0.045
Peer victimization	0.024^∗∗∗^	**0.024^∗∗∗^**	0.194^∗∗^	**0.160^∗∗^**	0.008^∗^	**0.009^∗∗^**
Mutual antipathy	–0.009	**–**	0.285^∗∗^	**0.249^∗∗^**	0.003	–
Peer rejection	0.003	**–**	–0.033	–	0.003	0.003
Sex^∗^PV	–0.013	**–**	–0.084	–	0.003	–
Sex^∗^MA	0.019	**–**	0.435^∗∗^	**0.555^∗∗∗^**	–0.009	–
Sex^∗^PR	0.008	**–**	0.099	–	0.007^∗^	**0.006^∗^**
Region	^∗^	**^∗^**	^∗∗∗^	**^∗∗∗^**	^∗∗∗^	**^∗∗∗^**
Valencia	0.006	0.040	4.361^∗∗∗^	**4.423^∗∗∗^**	–0.229^∗∗∗^	–**0.231^∗∗∗^**
Balearic	0.169	0.171	–0.169	–0.142	–0.106	–0.108
Andalusia	–0.111	–0.090	0.332	0.337	–0.372^∗∗∗^	–**0.375^∗∗∗^**
Castile-Leon^a^	0^a^	0^a^	0^a^	0^a^	0^a^	0^a^
$S_{u}^{2}$ (Classroom Intercept)	0.005	0.004	2.577	2.621	0.003	0.003

–2LL^b^	1563.162^∗∗∗^	**1579.049^∗∗∗^**	5528.775^∗∗∗^	**5530.981^∗∗∗^**	954.150^∗∗∗^	**955.349**^∗∗∗^
	13 Ps	7 Ps	13 Ps	10 Ps	13 Ps	10 Ps
Δ(-2LL): Fitted	**15.887^∗^**, 6 Ps		2.206, 3 Ps		1.199, 3Ps	
model – Full model						
AIC	1589.162	1593.049	5554.775	5550.981	980.150	975.349
Δ(AIC)	3.887		–3.794		–4.801	
Pseudo-*R*^2c^100^c^	10.05%	7.95%	21.16%	20.98%	15.13%	14.98%

*Significant results for the whole set and the results for each final equation appear in bold (Fitted model). ^*a*^Reference group: Castile-Leon region. ^*b*^The corresponding probability is calculated compared with its null model. ^*c*^Coefficient of determination, or percentage of the explained variance of the dependent variable by the independent variables. *^∗^p* < 0.05; ^∗∗^*p* < 0.01; *^∗∗∗^p* < 0.001.*

**FIGURE 1:**
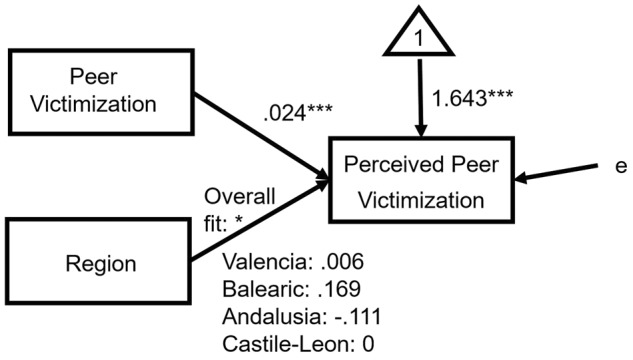
Effect diagram of M2 model, on **Table 2**, for raw data. ^∗^*p* < 0.05, ^∗∗∗^*p* < 0.001.

**FIGURE 2:**
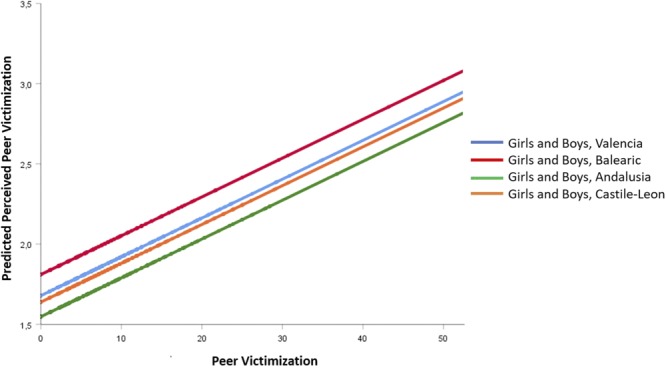
Forecasted values of Perceived Peer Victimization according to Peer Victimization, corresponding to M2 model on **Table 2**.

The -2LL shows that the fitted model M2 improved the null model significantly. According to the Δ(-2LL), there were significant differences between M1 and M2, but model M2 with fewer parameters was chosen because: (a) following Burnham et al. (2011), it cannot be confirmed that there is any difference between two nested models with a difference in AIC below 7, Δ(AIC) = 3.887; and (b) the difference in the percentage of variance explained of perceived peer victimization was very small (10.05% in M1 versus 7.95% in M2). The dummy variable region was significant on the whole (*p* < 0.05). The effect of Sex was not significant, thus the predictive equation of perceived peer victimization in M2 was the same for both boys and girls, for the reference region (Castile-Leon):

(1) $$\begin{array}{c} {\mathrm{PPV}}_{\left(\mathrm{Boys}\;\mathrm{and}\;\mathrm{Girls}\right)}^{\prime }\;=1.643\;+\;0.024\mathrm{PV}, \end{array}$$

with *p* < 0.001 for *B*_0_, and *p* < 0.001 for the coefficient *B*_1_, as can be observed in **Table 2**, effect diagram on **Figure 1** (Muthen et al., 2016), and forecasted values of perceived peer victimization on **Figure 2**. On **Figure 1**, dummy variables of more than 3 categories, by definition, would not co-vary within themselves or with any other IV (Region has 4 categories, with 3 dummies, so it does not covariate with the other IVs). The overall effect of Region, for their corresponding dummies, has a probability of 0.039 (^∗^*p* < 0.05).

Note that in the equations for the other three regions, the intercept changes but the slope does not (e.g., for Andalusia: PPV ′_(Boys and Girls)_ = 1.643 - 0.090 + 0.024⋅PV = 1553+0.024⋅PV); that is, they are parallel lines, but as the effect of Sex was not significant there is only one line by Region, representing values of both boys and girls.

#### Dyadic Perceived Disliking

**Table 2** shows that the ICC was small and the $S_{u}^{2}$ = 2.621 was nonsignificant. The significant antecedent variables of dyadic perceived disliking (full model M3) were: mutual antipathies, peer victimization, Sex^∗^Mutual antipathies, and region. In addition, because of the nesting principle in the interaction of variables (Jose, 2013), sex was also left in the fitted model (M4).

**FIGURE 3:**
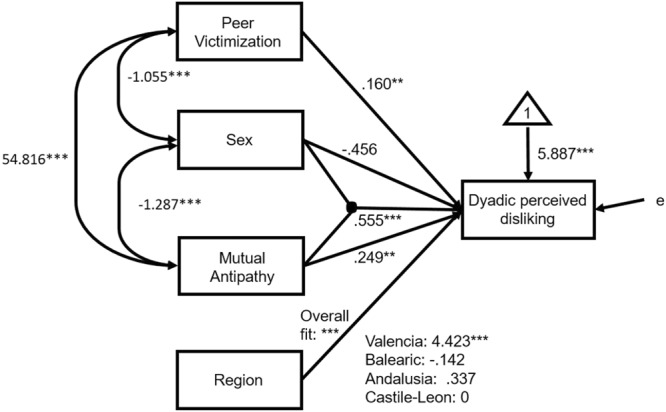
Effect diagram of M4 model, on **Table 2**, for raw data. ^∗∗^*p* < 0.01, ^∗∗∗^*p* < 0.001.

The fitted model M4 was significant on the whole to explain dyadic perceived disliking (the comparison between the -2LL of M3 and its null model produced a significant difference, *p* < 0.001). M4 explained 20.98% of the variance of dyadic perceived disliking. When M3 was compared with M4, no differences were found between the two, Δ(-2LL) = 2.206, *df* = 3, nonsignificant (ns). However, the AIC was lower in M4, and therefore, we chose the simplest model of the two, M4, represented by the effect diagram on **Figure 3**.

**FIGURE 4:**
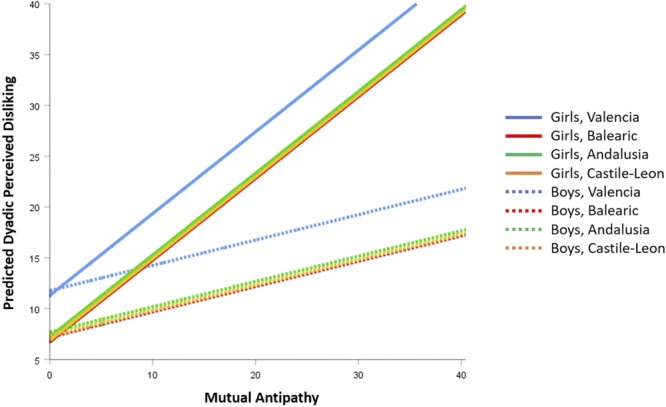
Forecasted values of Dyadic Perceived Disliking according to Mutual Antipathy, corresponding to M4 model on **Table 2**.

To respond to the question of how the interaction of Sex^∗^Mutual antipathies affects dyadic perceived disliking, the equations of forecasted values in **Figure 4** and **Table 2** were developed. With the average of peer victimization (9.103) as reference and Castile-Leon as reference Region, the expected values of dyadic perceived disliking for boys (Sex = 0) and girls (Sex = 1) were:

(2) $$\begin{array}{c} {\mathrm{DPD}}^{\prime }{}_{\left(\mathrm{Boys}\right)}\;=\;5.887\;-\;0.456\cdot 0\;+\;0.249\cdot \mathrm{MA}\;+\;0.160\cdot 9.103\;+\;0.555\cdot 0\cdot \mathrm{MA}\;=\;7.343\;+\;0.249\cdot \mathrm{MA} \end{array}$$

(3) $$\begin{array}{c} {\mathrm{DPD}}^{\prime }{}_{\left(\mathrm{Girls}\right)}\;=\;5.887\;-\;0.456\cdot 1\;+\;0.249\cdot \mathrm{MA}\;+\;0.160\cdot 9.103\;+\;0.555\cdot 1\cdot \mathrm{MA}\;=\;6.887\;+\;0.804\cdot \mathrm{MA} \end{array}$$

As seen in Equations (2) and (3) and **Figure 4**, the girls’ slope of dyadic perceived disliking (*B*_1,Girls_ = 0.804, *t* = 4.113, *p* < 0.001) as a function of mutual antipathies was significantly higher than the boys’ slope (*B*_1,Boys_ = 0.249, *t* = 7.827, *p* < 0.001) because the interaction coefficient was significant (difference of slopes: *B*_Intr_ = 0.555, *t* = 2.243, *p* = 0.025). **Figure 4** depicts the slopes for the 4 regions. The representations are parallel lines by Sex (Boys have a slope of 0.249, and Girls a slope of 0.804), with changes only to the *y*-intercept (Jose, 2013). The Valencian Region had the highest dyadic perceived disliking.

#### Loneliness

**Table 2** shows that the ICC was small and the $S_{u}^{2}$ = 0.003 was nonsignificant. The significant antecedent variables of loneliness (full model M5) were: peer victimization, Sex^∗^Peer rejection, and region. In addition, because of the nesting principle in the interaction of variables (Jose, 2013), sex and peer rejection were also left in the fitted model (M6).

**FIGURE 5:**
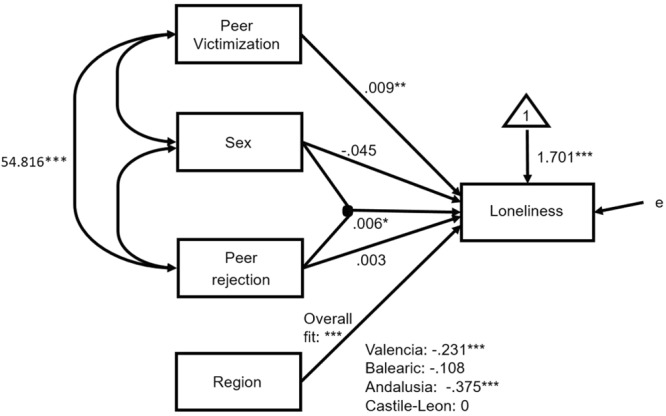
Effect diagram of M6 model, on **Table 2**, for raw data. ^∗^*p* < 0.05, ^∗∗^*p* < 0.01, ^∗∗∗^*p* < 0.001.

The fitted model M6 was significant on the whole to explain loneliness (*p* < 0.001). M6 explained 14.98% of the variance of loneliness. A comparison of M5 and M6 revealed no differences between the two, Δ(-2LL) = 1.199, *df* = 3, ns. Furthermore, AIC was lower in M6; consequently, we chose the simplest model of the two, M6, represented by the effect diagram on **Figure 5**.

**FIGURE 6:**
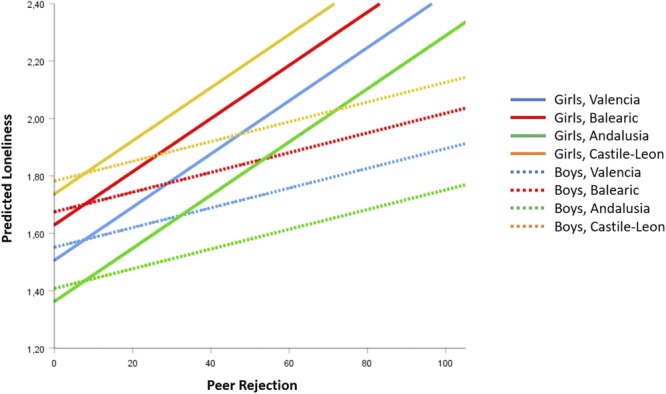
Forecasted values of Loneliness according to Peer Rejection, corresponding to M6 model on **Table 2**.

To respond to the question of how the interaction of Sex^∗^Peer rejection affects loneliness; the equations of forecasted values from **Figure 6** and **Table 2** were developed. Taking the average of peer victimization (9.103) and Castile-Leon region as references, the expected values of loneliness for boys (Sex = 0) and girls (Sex = 1) were:

(4) $$\begin{array}{c} L_{\left(\mathrm{Boys}\right)}^{\prime }\;=\;1.701\;-\;0.045\cdot 0\;+\;0.003\cdot PR\;+\;0.009\cdot 9.103\;+\;0.006\cdot 0\cdot PR=1.783\;+\;0.003\cdot PR \end{array}$$

(5) $$\begin{array}{c} L_{\left(\mathrm{Girls}\right)}^{\prime }\;=\;1.701\;-\;0.045\cdot 1\;+\;0.003\cdot PR\;+\;0.009\cdot 9.103\;+\;0.006\cdot 1\cdot PR=1.738\;+\;0.009\cdot PR \end{array}$$

As seen in Equations (4) and (5) and **Figure 6**, the girls’ slope of loneliness as a function of peer rejection (*B*_1,Girls_ = 0.009, *t* = 4.377, *p* < 0.001) was significantly higher than the boys’ slope (*B*_1,Boys_ = 0.003, *t* = 1.772, *p* = 0.076) because the interaction coefficient was significant (difference of slopes: *B*_Intr_ = 0.006, *t* = 2.125, *p* = 0.034). The region slopes are parallel lines by Sex. In the variable region, there were significant differences on the whole in the expected level of loneliness (*p* < 0.001), with the Castile-Leon Region having the highest level and Andalusia and Valencia the lowest.

## Discussion

As expected according to symbolic interactionism, adverse peer experiences were positively associated with negative interpersonal perceptions (Cillessen and Bellmore, 2004). We can conclude that the association between each adverse experience and a negative self-perception is affected by the presence of other negative experiences, and that each adverse experience is more forcefully linked to a negative perception in line with its specific nature and social level or focus, and that this association is stronger for girls.

### First Objective: Contribution of Adverse Peer Experiences to Each Negative Interpersonal Perception

#### Perceived Peer Victimization

The independent variable peer victimization had a significant influence on perceived peer victimization. At this age, our data support the broad consensus that perceived victimization is directly associated with being the target of aggressive behavior by other classmates (Boivin and Hymel, 1997). Our measures of peer victimization and perceived peer victimization both consisted of heterogeneous items (physical, verbal, and relational mistreatment), which may explain why sex had no influence on their relation (Bevans et al., 2013).

Given that peer rejection can be a precursor of peer victimization, and that some mutual antipathies might be related to situations of bullying (Card and Hodges, 2003, 2007), a connection is to be expected between these adverse experiences and perceived victimization. However, our data show that, in the presence of peer victimization, peer rejection and mutual antipathies are not associated with feelings of being victimized. In other words, as Hymel et al. (1999) concluded, perceived peer victimization only occurs through manifest behaviors of aggression and exclusion.

#### Dyadic Perceived Disliking

As expected, the principal antecedent of dyadic perceived disliking was mutual antipathies. This result is in agreement with Card’s (2010) statement that an unpleasant dyadic relationship such as mutual antipathy may imply a particularly intense feeling of social rejection. The fact that antipathetic relationships are reciprocal and interdependent makes rejection more obvious and rejecters more easily identifiable, and therefore, these relationships are experienced with greater emotional intensity (Sandstrom and Zakriski, 2004). Our results reinforce the interpretation of mutual antipathies as a sense of loss of relationships that provide social and emotional support (Card and Hodges, 2003).

The interaction of mutual antipathies with sex suggests that girls are more responsive to dyadic relationships than boys are. The types of relationships in which boys and girls engage offer a valid explanation of this result. Girls generally pay more attention to and place more importance on dyadic relationships than boys (Rose and Rudolph, 2006). Paradoxically, girls’ greater concern about friendships increases their fragility and the likelihood that they may feel threatened and rejected in a relationship (Culotta and Goldstein, 2008). Hence, mutual antipathies, which, in some cases, derive from a previous friendship, would have a greater impact on the dyadic perceived disliking among girls as a consequence of the dissolution of the reciprocal positive interaction (Vanhalst et al., 2013). Indeed, Muñoz-Tinoco (2015) found that the number of friendships that turned into mutual antipathies during the first school year was higher in girl dyads than in boys.

The influence of peer victimization on dyadic perceived disliking could be due to the fact that four-fifths of victimized children are rejected by their peers (Schuster, 2001). Rejected children who are victimized are more aware of their peers’ rejection than rejected children who are not victimized (Sandstrom and Zakriski, 2004) because victimization makes them understand that they are treated negatively because the others do not like them (“If someone hurts me, it is because they do not like me”). However, peer rejection, when examined together with the other two experiences, is not linked with dyadic perceived disliking. We could venture two reasons why peer rejection does not entail greater dyadic perceived disliking. First, peer rejection is essentially a covert negative evaluation, not an explicit behavior against another individual (McDougall et al., 2001; Leary, 2005). Second, some children, particularly rejected children, tend to overestimate their social competence compared to their peers’ view (White and Kistner, 2011) and underestimate the rejection they receive, which can act as a protector for the rejected children (Blackhart et al., 2009). Both explanations imply a difficulty to identify the rejecters, or dyadic perceived disliking.

#### Loneliness

Peer rejection is a specific antecedent variable of loneliness. The group nature of peer rejection (García Bacete et al., 2010) and the strong influence of social and group factors on rejection (Mikami et al., 2010) can explain why feelings of loneliness are predicted by situations of peer rejection. Our results also suggest that this association is stronger among girls. Paquette and Underwood (1999) observed that girls were more sensitive to social situations involving peer rebuff, and McDougall et al. (2001) reported that girls are most likely to react to peer rejection and peer victimization with isolation and loneliness.

We also found a positive association between peer victimization and loneliness. This result could be explained by the dynamics of bullying that make victims feel that nobody can help them and that they may lose their friends (Catterson and Hunter, 2010). However, unlike previous findings, when the other adverse experiences are present, our results do not support that mutual antipathy is linked to loneliness. Although mutual antipathy relationships tend to aggravate other adverse experiences, they usually have less impact on loneliness (Vanhalst et al., 2013) because, beyond the conflict with their respective enemy, each of the parties is likely to be accepted by other group members. These group members sometimes even become sources of support or allies against the other party (Card and Hodges, 2003).

### Second Objective: Sex Differences in the Association Between Adverse Peer Experiences and Negative Interpersonal Self-Perceptions

Another important result concerns the interactions of sex with adverse experiences. The peer socialization model in school, which contributes to the development of sex-typed peer motivations and interactions (Rose and Rudolph, 2006), can support theorizing on possible differences in the association between adverse peer experiences and negative interpersonal perceptions. Research indicates that girls value friendships, hold communal goals, and gain more emotional benefit from relationships than boys do, and that, in contrast, boys value strength and dominance, and have more self-interested goals in their peer relationships (Rose and Rudolph, 2006; Rose and Smith, 2009). Our results are coherent with this model. On the one hand, girls are more aware of peer rejection and mutual antipathies than boys, which may be because of their more communal and friendship goals, whereas boys are more responsive to peer victimization, which may be because of the difference of power involved. On the other hand, although the frequency of adverse peer experiences is greater among boys than among girls, the results do not show a stronger influence of adverse peer experiences on negative interpersonal perception for boys. On the contrary, the associations between self-perceptions and experiences are higher in girls than in boys, particularly in the dyadic level, which may indicate that it is not only reality that has an impact on the perception, but also the interpretation that each sex gives to the context of the relationship (Salmivalli and Isaacs, 2005).

### Influence of Classroom and Region

We used as control variables the sampling variables classroom (35 classrooms) and region (4 regions). Despite the high number of classes, the random effect of class was nonsignificant, which indicates that there is no difference between classes. However, the students presented differences in their interpersonal perceptions according to the region in which they live, but the influence of each region is different in each interpersonal perception. The factor region globally influences perceived peer victimization but with no significant differences between regions: whereas the students in Valencia reported the most dyadic perceived disliking, and the children of Andalusia reported the least loneliness. Of these three results, the one referred to loneliness may be explained because in Andalusia, there is a tradition of great extroversion with everybody, relatives, friends, acquaintances and strangers, celebration of frequent street festivals, and even religious celebrations have a socializing and festive component, which promotes associationism and integration. The results concerning perceived peer victimization and dyadic perceived disliking would require a detailed study to deepen it.

## Conclusion

Our data confirmed that there is a significant positive association between adverse peer experiences and negative interpersonal self-perceptions, yet we have also seen that each type of peer relationship influences mostly the interpersonal self-perception focused on the same peer system level. Furthermore, this study, according to our fourth hypothesis, grants support to Hymel et al.’s (1999) contention that children infer how well they get along with peers mostly through the social behaviors peers direct toward them. The fact that children at this age rely primarily on behavior to build their interpersonal perceptions can be explained by their barely incipient ability for interpersonal perspective and social self-awareness (Bellmore and Cillessen, 2003).

### Contributions

A general theoretical implication from our findings is that research should take into account the complexity of the social peer system, as already suggested by Zhang et al.’s (2014) study. A more specific theoretical implication refers to the findings in mutual antipathy and children’s dyadic negative cognitions, which highlight the relevance of dyadic relationships in children’s daily experiences (Sandstrom and Zakriski, 2004; Muñoz-Tinoco, 2015; Daniel et al., 2016). It seems that, at this age, a measure for negative dyadic peer experiences using active negative reciprocity is more useful than the absence of positive reciprocity, like friendlessness, used in previous studies (Boivin and Hymel, 1997; Ladd et al., 1997; Salmivalli and Isaacs, 2005).

At the individual level, we found sex differences in the perceptions of adverse dyadic and group experiences. Girls perceive interpersonal experiences more globally than boys. Despite undergoing fewer negative peer experiences than boys, they grant a greater value to these adverse experiences and consider them as lost opportunities for satisfying their social needs for closeness, acceptance, and friendship (Vuijk et al., 2007).

### Limitations and Directions for Future Research

This study used a standard cross-sectional methodology. Even though it is an established methodology in behavioral sciences, it shows limitations. Future research using longitudinal designs across the elementary-school years could help to understand better the long-term influence of adverse peer experiences on interpersonal perceptions. Although we used a large number of variables of different levels, the reality is much more complex, and we did not consider the wide heterogeneity among rejected children (García Bacete et al., 2010), among victimized children (Juvonen and Graham, 2014), and between antipathetic dyads (Card, 2010). It would be interesting to include covert rejection forms along with more visible ones, to differentiate between physical and relational victimization or between enemies with or without friends. In the same line, it would be of interest to contrast the positive results found in mutual antipathies with studies simultaneously including measures of friendlessness. The inclusion of friendlessness would help to confirm our explanations about the absence of some expected results.

Another limitation is that our research focused on the influence of social reality on social cognitions, but the inverse process exists simultaneously (Cillessen and Bellmore, 2004). Our results of sex differences support the latter perspective. Girls, despite being subjected to fewer negative experiences, showed a greater tendency to identify rejecters and perceive themselves as lonely. These sex differences show that not only the objective reality of experiences shapes our perceptions, but also our awareness of and cognitions about these experiences. On the contrary, we may have failed to obtain some significant association between adverse peer experience and interpersonal perceptions because of our lack of consideration of this second perspective. For instance, the cognitive biases of rejected children to underestimate their rejection might explain why peer rejection was not associated with dyadic expected rejection, as could be expected (MacDonald and Cohen, 1995).

### Applications for Educational Practice

These results point out the importance both of the scenarios in which peer experiences take place and the construction that students make of these experiences, and show that interventions in schools should cover all the levels of the peer social system. Research emphasizes the importance of working in the ecology of the class (Serdiouk et al., 2013) and that the intervention activities should be applied by the teachers themselves (Durlak et al., 2011). But to do this, teachers need to know and use in their daily practice instruments for evaluating peer relationships, such as those used in this study, and increase their training on how students relationships are (Gest and Rodkin, 2011), how they interact themselves with students (O’Connor et al., 2011), and the influence of their classroom practices on classroom climate (Serdiouk et al., 2013).

Mutual antipathy was associated with dyadic perceived disliking, which may lead to the idea that we should intervene to eradicate this kind of adverse peer experiences. However, mutual antipathy may have an adaptation function (Card, 2010; Daniel et al., 2016), and therefore, it is not to be eliminated *a priori*. Furthermore, we know that the antipathy ties associated with more negative outcomes are the ones that are stable, asymmetrical, and numerous. Consequently, the first recommendation is that teachers should allow children to express their antipathies. Only by knowing these antipathy relationships and observing their evolution would teachers know which ones are “useful” for the child’s adjustment and which ones might be harmful.

Our study contributes to sensitize teachers about the greater responsiveness and consequent maladjustment of girls to adverse experiences. Girls at this young age do not yet require much or evident behavioral abuse by their peers in order to feel or interpret negative social experiences as losses, with the consequent impact on their development/adjustment. Additionally, given that girls show a better school behavior and achieve higher academic performance than boys, there may be a risk that girls’ negative social exchanges be hidden and therefore not intervened on. Furthermore, even when an intervention does take place, teachers’ beliefs about the appropriate strategies that children should use to face negative peer experiences make differences between boys and girls. While boys should implement active strategies, girls are encouraged to avoid conflict situations (Troop-Gordon and Ladd, 2015). These findings are relevant for educational practice in that teachers could be more aware that girls and boys react differently in adverse situations, and how their own beliefs and practices might contribute to a worse adjustment by discouraging girls from tackling actively adverse peer experiences.

Our findings could be useful for teachers to design classroom contexts in which, on the one hand, children are given opportunities to interact cooperatively with peers (Mikami et al., 2010), and take advantage of the quality of dyadic interactions (García Bacete et al., 2013), in order to know each other better, promote the coordination of perspectives and foster empathy and prosociality (Domitrovich et al., 2017). This would endear peers to the rejected children, for instance, and refrain rejected children from having to make friends with each other, as it often happens (Huitsing et al., 2012). Such supportive teachers and classrooms provide all children with social-emotional skills and resources to help them tackle rejection, victimization, and mutual antipathies (Longobardi et al., 2018). On the other hand, not only should these contexts help children express their positive opinions, but children should also learn how to express negative judgments in an authentic and assertive way (Saarento et al., 2013). Teachers should encourage these negative but assertive comments, and avoid creating artificial contexts where the standard of the class is “everyone should get along with everyone and everyone is friend with everyone.” By doing so, children can become aware of their rejecters or enemies and bullies and react assertively. Moreover, the overt expression of the rejected children’s feelings would facilitate the rejecters’ or bullies’ putting themselves in the place of the rejected and victimized child (Calhoun, 2011; McQuade et al., 2012), and empower active bystanders to fight against peer mistreatment (Sandstrom et al., 2017).

## Author Contributions

FGB: led and designed the study, coordinated data collection, contributed to the analysis and interpretation of the data, and drafted the manuscript. IS-G, VM-T, and IJ-L: contributed to data collection, revised critically the study, and participated in drafting and editing the manuscript. GMP: contributed to data collection, and participated in interpreting the data and editing the manuscript. JR: performed the analysis and interpretation of the data, and participated in drafting the manuscript. All authors approved the final manuscript as submitted.

## Conflict of Interest Statement

The authors declare that the research was conducted in the absence of any commercial or financial relationships that could be construed as a potential conflict of interest.
